# A 316MP, 120FPS, High Dynamic Range CMOS Image Sensor for Next Generation Immersive Displays †

**DOI:** 10.3390/s23208383

**Published:** 2023-10-11

**Authors:** Abhinav Agarwal, Jatin Hansrani, Sam Bagwell, Oleksandr Rytov, Varun Shah, Kai Ling Ong, Daniel Van Blerkom, Jonathan Bergey, Neil Kumar, Tim Lu, Deanan DaSilva, Michael Graae, David Dibble

**Affiliations:** 1 Forza Silicon (AMETEK Inc.), Pasadena, CA 91107, USA; jatin.hansrani@ametek.com (J.H.); sam.bagwell@ametek.com (S.B.); oleksandr.rytov@ametek.com (O.R.); varun.shah@ametek.com (V.S.); kailing.ong@ametek.com (K.L.O.); vanblerkom@gmail.com (D.V.B.); jonathan.bergey@ametek.com (J.B.); neil.kumar@ametek.com (N.K.); tim.lu@ametek.com (T.L.); 2 Sphere Entertainment Co., New York, NY 10121, USA; deanan.dasilva@sphereentertainmentco.com (D.D.); michael.graae@sphereentertainmentco.com (M.G.); ddibble@msg.com (D.D.)

**Keywords:** CMOS image sensor, high dynamic range, stitched image sensor, single exposure dual gain, large format image sensor, immersive displays, cinematography, high-speed imager, low-noise image sensor, high frame rate, rolling shutter, ADC counter distribution, high-speed clock distribution, horizontal smearing

## Abstract

We present a 2D-stitched, 316MP, 120FPS, high dynamic range CMOS image sensor with 92 CML output ports operating at a cumulative date rate of 515 Gbit/s. The total die size is 9.92 cm × 8.31 cm and the chip is fabricated in a 65 nm, 4 metal BSI process with an overall power consumption of 23 W. A 4.3 µm dual-gain pixel has a high and low conversion gain full well of 6600e- and 41,000e-, respectively, with a total high gain temporal noise of 1.8e- achieving a composite dynamic range of 87 dB.

## 1. Introduction

In this paper, we present a very large 2D-stitched, high-frame-rate, 316MP CMOS image sensor capturing video at 18 K × 18 K resolution. We employ a single-exposure dual-gain HDR approach that inherently mitigates motion artifacts that may appear in other multi-exposure HDR techniques [1,2]. The fundamental challenge of this sensor design is to read out a physically large 2D pixel array at a high frame rate while maintaining a good noise performance.

Large-format, next-generation immersive displays provide challenging requirements for video capture: the combination of the size and resolution of the display that necessitates the detailed image resolution also clearly exposes any deficiencies in the image. This requires a sensor that will create very-high-resolution imagery while maintaining image quality, low noise, high dynamic range, and minimal shutter/image artifacts [3].

One approach for generating high-resolution content is to stitch together multi-camera array video footage in post-production to generate a super-resolution video. Video processing involved for this is algorithmically challenging, computationally intensive for post-production workflows [4], and often creates artifacts such as ghosting, blur, and discontinuity at the stitch seams caused by parallax between input views [5].

To overcome these issues, in this paper, we present a very large 2D-stitched 316MP CMOS image sensor capturing video at 18 K × 18 K resolution. This rolling shutter sensor operates in either a high-frame-rate single-gain readout mode or a reduced-frame-rate HDR mode. The HDR mode leverages the dual-gain capability of the pixel to allow extended dynamic range within a single exposure, mitigating motion artifacts that might appear in other HDR approaches.

## 2. Readout Architecture

**Figure 1:**
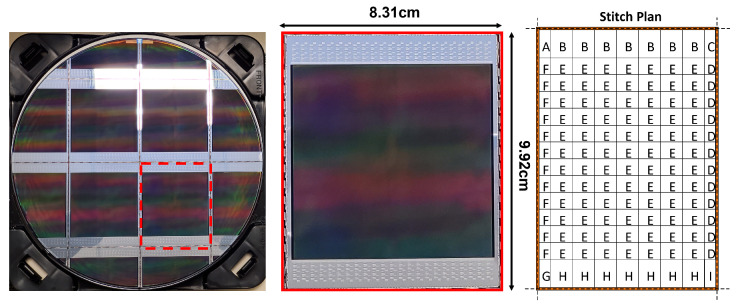
Sensor on a 12 inch wafer (4 dies per wafer), die photo, and stitch plan.

**Figure 2:**
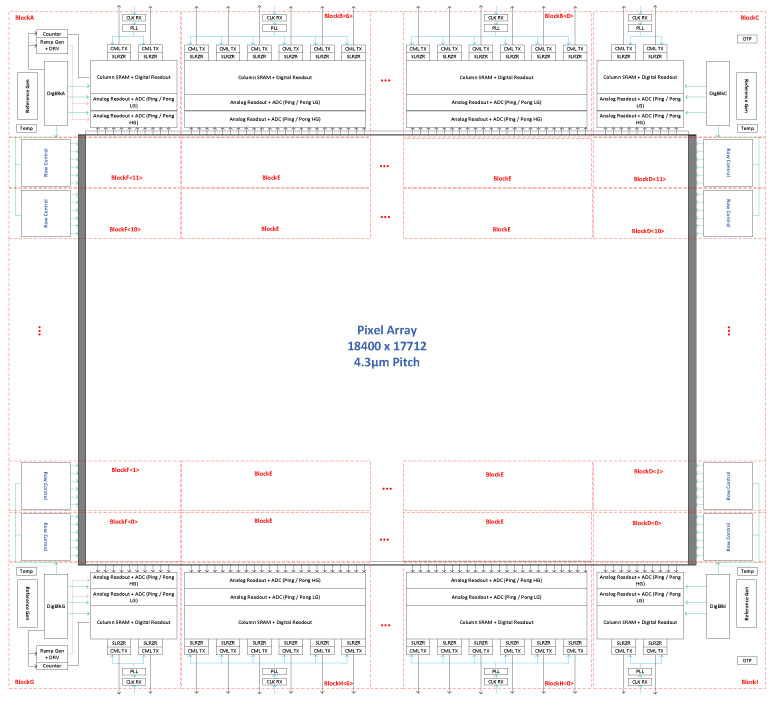
Detailed block diagram showing sensor partitioning.

The sensor stitch plan (Figure 1) has a total of 8 vertical and 13 horizontal stitch lines. The top and bottom halves of the pixel array are read out independently, with each half sampling 6 pixel rows at a time. To meet the high frame rate requirement, the sensor timing is pipelined with overlapping pixel sampling, ADC conversion, and digital readout phases. Figure 2 shows a detailed sensor block diagram with more details of the sensor partitioning.

The sensor readout is divided into repeating units. Stitch blocks ‘B’ and ‘H’ consist of six readout units each, whereas stitch blocks ‘A’, ‘C’, ‘G’, ‘I’ contain two readout units each (Figure 2). Every readout unit is responsible for sampling (400 pixel columns × 6 pixel rows) per ‘row time’. Each pixel column feeds into a set of 12 single slope ADCs (6 rows × ping/pong), so there are a total of 441,600 ADCs in this design. To fit in the 4.3 µm pixel pitch, each pixel column has 4 vertically stacked rows of 3 ADCs arrayed at a 1.4 µm pitch.

## 3. Stitch Plan

As with any stitched design, it is important to co-design the sensor architecture along with the stitch plan to account for the required repetition of large areas of the design during stitching [6,7]. Because of the extreme physical size of this design, and thus the large amount of repetition, there was an unusually complex planning process to make sure the architecture and stitch plan satisfied all of the mechanical, optical, electrical, and process requirements.

**Figure 3:**
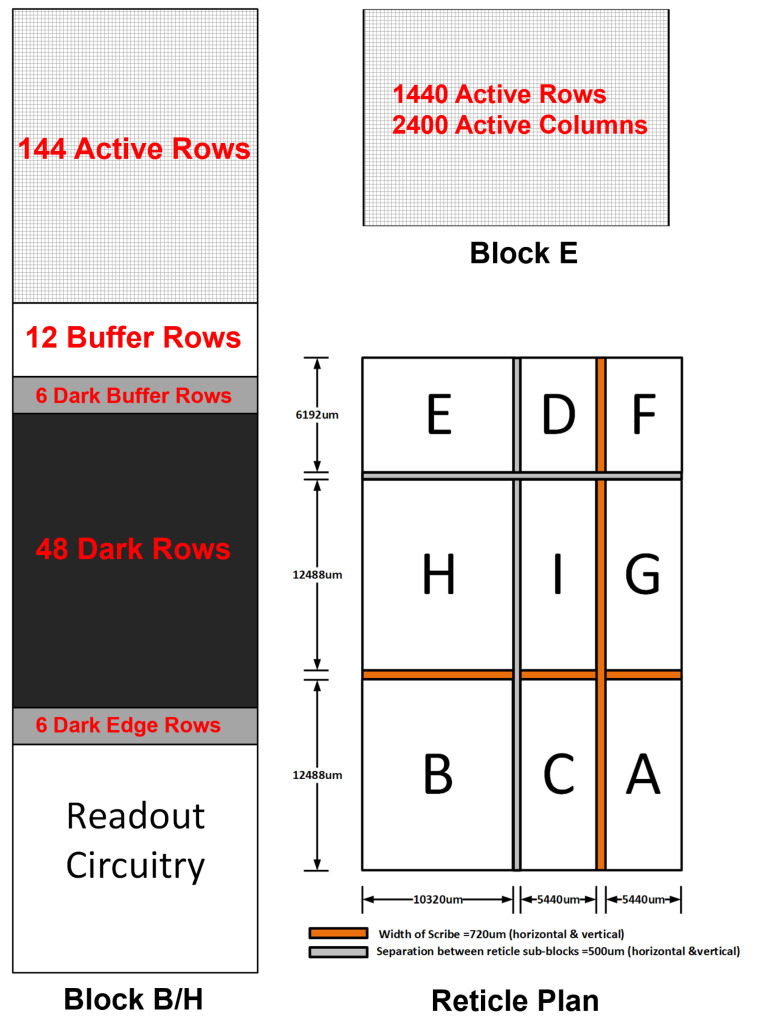
Distribution of active and dark rows in block B/H, block E, and final reticle plan.

As shown in the reticle plan (Figure 3), we have reticle blocks B and H, each containing the column parallel readout circuitry, including single-slope ADCs, analog SRAM for data aggregation, digital logic for data assembly and formatting, a clock receiver, PLL, a high-speed clock distribution network, serializers, and CML output drivers. In addition, optically black rows (dark rows) and active rows (if any) also need to be accommodated in blocks B and H. Based on the available IP and the readout architecture, having a split readout reading six rows at a time per side with ping/pong ADCs, the heights of block B and H were first estimated.

With height estimates of block B and H, given the maximum reticle height and minimum required width of the horizontal scribe line, the maximum available height of block E was calculated. With the objective of minimizing the total number of horizontal stitch lines (to reduce any image artifacts caused by stitching), knowing the maximum height of block E allowed us to calculate the maximum active pixel rows that can fit in block E. However, some architectural constraints (e.g., a non-integer pixel pitch of 4.3 µm, split readout, and six row reads per side with ping/pong ADCs) and fabrication rules (e.g., all reticle block dimensions must be 1 µm multiple) further limit the maximum allowable active pixel rows in block E. Satisfying all these constraints and the specification for the total number of active rows, it was decided that each block E would contain 1440 active rows, vertically stitched 12 times. The remainder of the 288 active rows and 96 dark rows were adjusted equally between block B and H.

The minimum width of reticle blocks D and F each containing row decoders, row logic, and row driver circuitry was primarily determined by the size of layout of these blocks. Additionally, the routing space allocation for bringing power from multiple supply domain pads and distributing it efficiently in the vertical direction (with only one available thick metal) needed to be carefully considered as well. Optically black columns (dark columns) and additional active columns (if any) also needed to be adjusted in blocks D/F.

The corner blocks A, C, G, and I contain peripheral circuits, such as a ramp generator, ADC counter, reference/bias blocks, temperature sensors, and digital blocks (for frame timer operations). These circuits could easily fit within the height and width set by blocks B/H and blocks D/F, respectively. In fact, there was additional room to fit readout circuitry for reading dark columns and extra active columns (not covered by block E/B/H repetition in the horizontal direction).

The maximum reticle width, minimum width of the vertical scribe line and required width of blocks D and F set an upper bound on the widths of blocks E/B/H, in effect determining the maximum number of active columns that can fit in block E.

Based on the pixel array size, frame rate, ADC resolution, data overhead, and maximum data rate per CML port, we calculated the total number of output data ports required to be 92 (46 per side). We partitioned the overall readout circuitry into identical repeating units called readout cores, each containing one serializer and CML output driver. The maximum number of pixel columns that can fit in a readout core were primarily determined by the maximum readout speed of the analog SRAM, which in turn is limited by the total RC parasitics on each bit line and readout sense amplifier offset voltage. Finally, fulfilling other constraints such as each block dimension being 1 µm multiple and an integer number of readout cores in blocks B and H, it was decided that each block E would contain 2400 active columns, with blocks B and H containing 6 readout cores serving 400 active columns each.

Blocks E/B/H were repeated 7 times horizontally to generate 16,800 active columns, and the remaining 1200 active columns and 368 dark columns were divided equally between blocks A, C, G, and I. Blocks A/C/G/I each contains two readout cores, with one readout core completely reading active columns and the other readout core reading half active columns and half optically black and buffer columns. The pixel distributions in blocks B/H and block E, along with the final reticle plan, are illustrated in Figure 3.

## 4. Operating Modes

**Figure 4:**
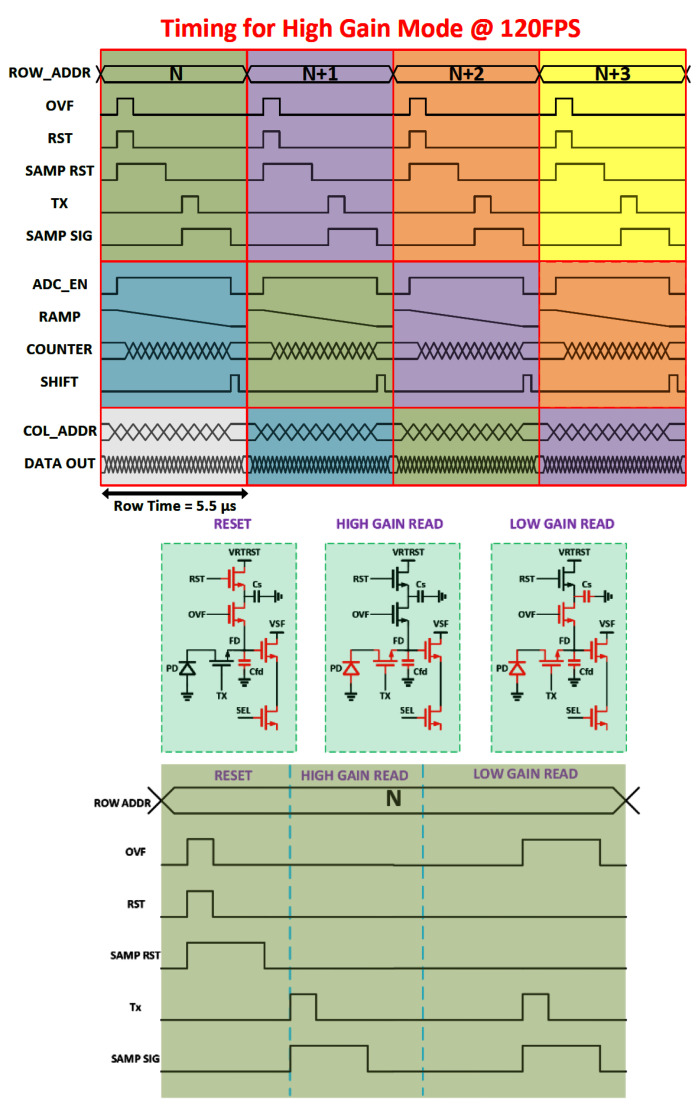
Sensor timing for single (high-gain or low-gain) gain operation.

**Figure 5:**
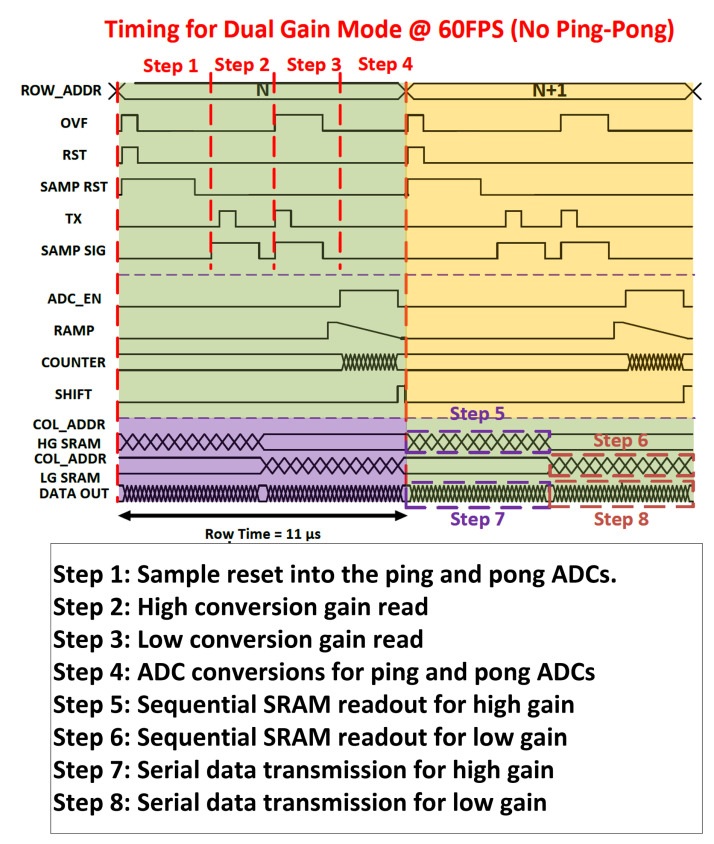
Sensor timing for single-exposure dual-gain (HDR) operation.

There are two modes of operation in this sensor: single-gain and dual-gain (HDR) mode. Die size limitations constrained the height of the readout circuitry, making it necessary to use the same ADC stack for both readout modes of the sensor. Detailed sensor timing for the single- and dual-gain modes is illustrated in Figure 4 and Figure 5.

### 4.1. Single-Gain Mode@120FPS

Every pixel was sampled with a fixed conversion gain (either high or low conversion gain) with a standard 4T read timing. The ADC array operated in a ping-pong type timing, where half the array was sampling while the other half was converting the previous sample.

### 4.2. Dual-Gain Mode@60FPS

To implement a single-exposure HDR functionality, we leveraged the existing programmable conversion gain of the pixel, where the high conversion is set by the floating diffusion (FD) capacitance and the low conversion gain achieved by connecting a secondary capacitor in parallel with the FD. The effective extended dynamic range is set by the capacity in the low-gain mode and the noise of the high-gain mode. Each integration is read out in both high- and low-conversion gains, and the resulting two values are transferred off-chip and processed into a single HDR value capturing more scene contrast.

#### 4.2.1. Proposed Dual-Gain Timing

Whereas the single-gain mode uses the ADC array in a pipelined ping-pong-type operation, the dual-gain mode re-configures the ADC array such that the ‘ping’ ADCs sample the high-gain pixel readout and the ‘pong’ ADCs sample the low-gain pixel readout, and all ADCs convert at once. This effectively doubles the readout time in this mode and thus halves the frame rate. The first operation in this readout is to reset the pixel FD and sample that reset value into both the high-gain and low-gain ADCs. Then, there is a first charge transfer in the pixel onto the FD node, and this value is read into the high-gain ADCs. Finally, the low-gain capacitor is switched into the pixel and another charge transfer is conducted (combining what was on the FD with any charge left in the photodiode), and this value is read into the low-gain ADCs. All ADCs are then converted simultaneously.

#### 4.2.2. Pros and Cons

The approach implemented here provides substantially less dynamic range improvement than the well-known LOFIC approach [8,9,10]. However, this approach allows for the simple re-use of the existing single-gain ADC array, whereas typically the low-gain readout from a LOFIC pixel is in the opposite polarity from the high-gain readout, necessitating modifications to the ADC sampling network and ramp. Due to the already extreme density of the ADC array and the readout height limitations, it was not possible to support a LOFIC-type readout in addition to the high-frame rate single-gain readout. This approach also obviated concerns about overflow path control and process tuning to better achieve first silicon success. One potential issue with sampling the reset level simultaneously for both high- and low-gain ADCs for the HDR operation mode is not being able to compensate for the charge injection from OVF while sampling the low-gain signal affecting the black level uniformity for the low-gain read [11]. Due to a very tight timing budget to achieve a 60 FPS maximum HDR frame rate (11 µs row time), we do not have the luxury of sampling the reset first into low-gain ADC followed by high-gain ADC. Based on post-layout pixel line settling simulations, we need an additional 2 µs of row time to separate out the reset sampling events for high- and low-gain ADCs. Therefore, for lower frame rates (<50 FPS for HDR), we can effectively mitigate the charge injection due to the pulsing OVF signal.

### 4.3. Analog SRAM Data Aggregation and Readout Order

The analog SRAM data aggregation needs to be compatible to support both single- and dual-gain HDR modes of operations in the sensor. In the single-gain mode using ping/pong ADC timing, only 6 ADCs convert in a row-time, and therefore, vertically stacking 6 SRAM banks within an ADC column would have been sufficient. However, in the dual-gain HDR mode both ping and pong (which are reconfigured as high-gain and low-gain ADCs, respectively) convert in the same row time. Hence, to remain compatible with both modes of operation, it was decided to vertically stack 12 SRAM banks within an ADC column.

The readout of the SRAM was carried out sequentially with reading one SRAM column per cycle, and therefore, to efficiently utilize data output bandwidth, the ADC latch outputs (from same ADC type) from two adjacent pixel columns were routed to the same SRAM column. With this approach, in the single-gain mode, every alternate SRAM column can be skipped when performing SRAM readout. In the dual-gain HDR mode, all high-gain ADC outputs are saved in one SRAM column and all low-gain ADC outputs are saved in the adjacent SRAM column. Therefore, all SRAM columns are read serially in the HDR mode.

All 6 (row) × 2 (column) high-gain and low-gain data are read sequentially, and hence, the memory requirement for an off-chip frame buffer storing high- and low-gain values for subsequent HDR processing is significantly reduced.

#### 4.3.1. Single-Gain Mode

**Figure 6:**
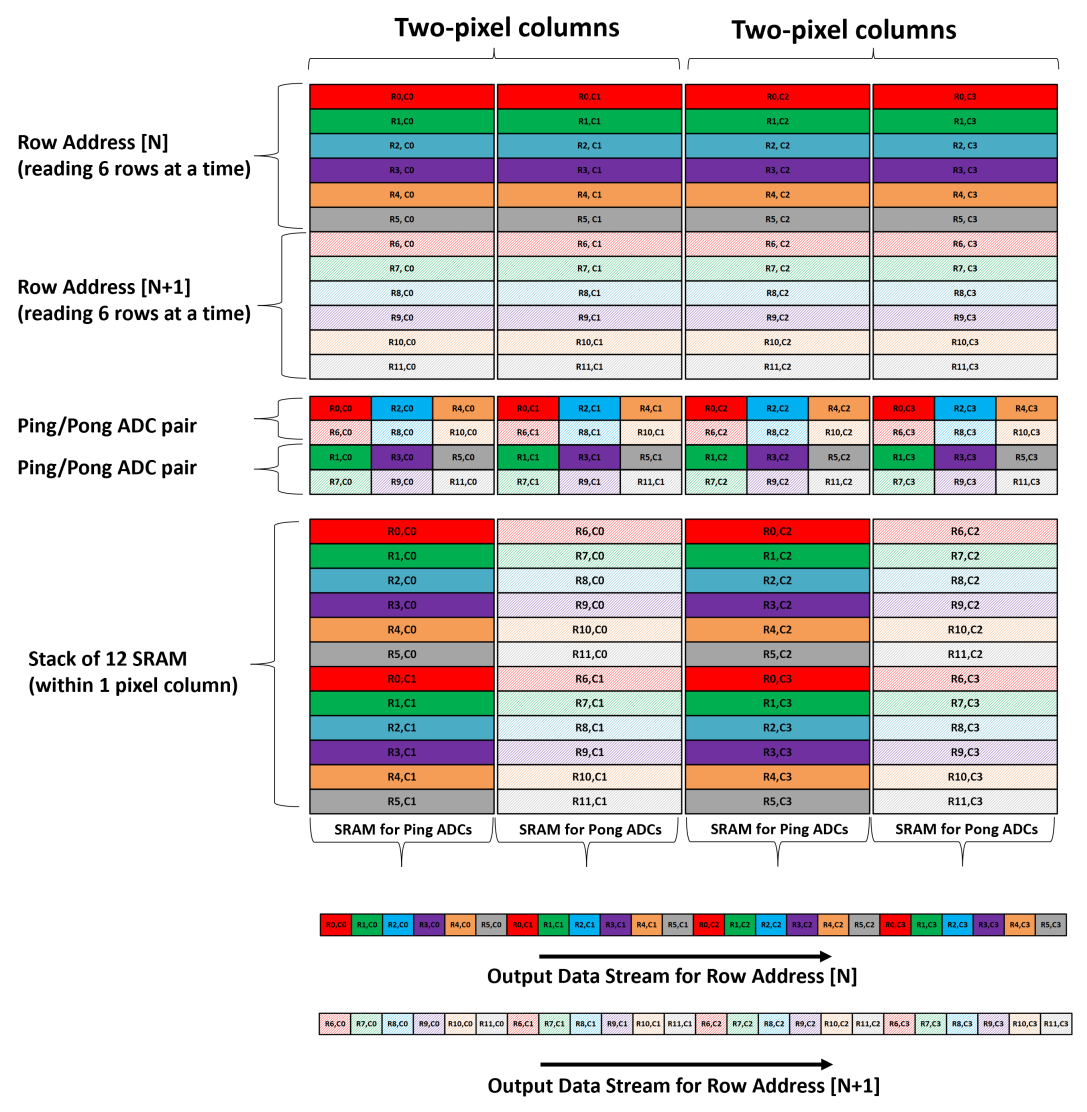
Data aggregation and readout order for single-gain mode.

Single-gain mode uses ping-pong timing in the ADC readout to increase the readout speed. As illustrated in Figure 6, Row[N+1] is sampled to a group of pong ADCs, while Row[N] data is converted in another group of ping ADCs. Even and odd row addressed pixels then effectively use different ADC groups (ping for even and pong for odd). Each ADC (ping or pong) has its own separate latch output, even though only one of them will be enabled for a particular row time (depending on whether ping/pong ADC is converting). Therefore, the SRAM bank could be shared between ping and pong ADCs for single-gain mode; however, for dual-gain mode (HDR), due to the fact that ping ADCs are configured as high-gain ADCs and pong ADCs are configured as low-gain ADCs, a separate SRAM bank is needed for high- and low-gain ADCs.

In the single-gain mode, for two adjacent pixel columns, the outputs of all ping ADCs are stored in the even SRAM column, and outputs of all pong ADCs are stored in the odd SRAM column. For Row[N] and Row[N+1], all even and odd SRAM columns, respectively, are read sequentially (Figure 6).

#### 4.3.2. Dual-Gain Mode

Dual-gain mode does not use ping-pong timing and instead reconfigures the ping ADCs to read the high-gain value and the pong ADCs to read the low-gain value from the pixel. One row readout operation (6 rows) utilizes all the available ADC bandwidth. The high- and low-gain values are sampled from the pixel into ADCs in quick succession; then, both the high-gain and low-gain ADCs are converted simultaneously.

**Figure 7:**
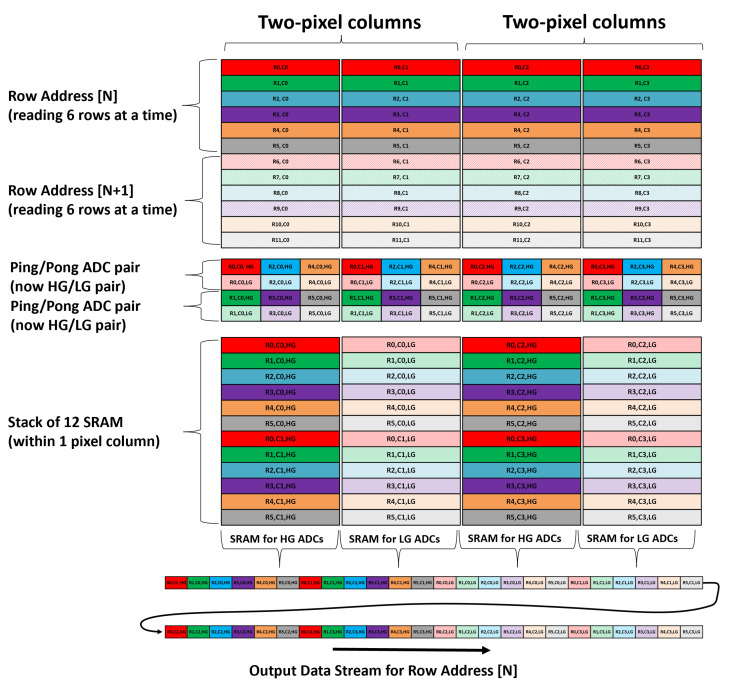
Data aggregation and readout order for dual-gain mode.

From the two adjacent pixel columns, the outputs of all the high-gain ADCs are stored in the even SRAM column, while the outputs of all low-gain ADCs are stored in the odd SRAM column. For Row[N], all SRAM columns are read sequentially, with even SRAM columns fetching the high-gain values and odd SRAM columns fetching the low-gain value from a 2 (column) × 6 (row) pixel sub-array (Figure 7).

## 5. Challenges and Solutions

We present some of the key challenges in designing this large-footprint CIS chip along with our proposed solutions.

### 5.1. ADC Electrical Crosstalk

Due to the vertical stacked arrangement of ADCs, the outputs of the top ADCs and pixel output lines travel the full height of the ADC column. The ADC layout is made at a small pitch of 1.4 µm with limited metal layers for shielding. This results in an unavoidable parasitic coupling between the pixel line (victim) and ADC outputs (aggressor), causing electrical crosstalk.

Other methods reported in the literature, such as the use of ’return-to-zero’ codes for the digital outputs [12], are primarily useful for clocked cyclic ADCs and do not work well for SS-ADC architectures having a continuous time comparison. Our choice in selecting the SS-ADC architecture for this CIS chip was primarily dictated by the large-format array, where we believe that using a cycling ADC would be much more challenging with the need to manage high switching transients and crosstalk/kickback on distributed reference levels. Continuous time SS-ADC is the simplest and therefore scales the easiest to extreme-size arrays.

**Figure 8:**
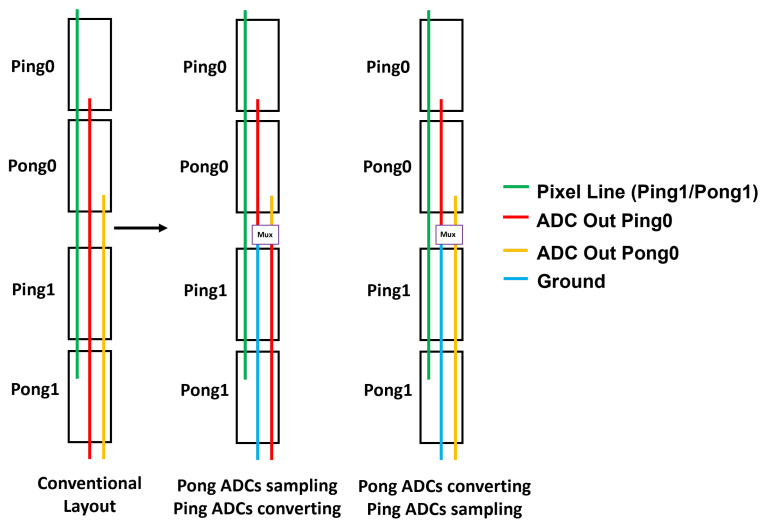
ADC output multiplexing network for electrical crosstalk mitigation.

In order to mitigate this electrical crosstalk issue, we implemented a novel shielding scheme by dynamically configuring the vertical ADC routing and shielding network according to the timing and mode of operation (as a means to reduce crosstalk). The ADC output multiplexing scheme is illustrated in Figure 8.

#### 5.1.1. Single-Gain Mode

Overlapping the sampling and ADC conversion phases (ping-pong timing) results in electrical crosstalk. In a large sensor like this, there can be a significant intensity difference between two six-row groups of twelve adjacent rows, resulting in ADC crosstalk, which is distinctly visible in an image. To mitigate this crosstalk, the outputs of the top two ADCs are multiplexed at the end of the second ADC. This frees up an additional routing line, which is used as a shield to reduce crosstalk. The simulated ADC crosstalk improvements as a result of this multiplexing scheme for various scenarios is summarized in Table 1.

#### 5.1.2. Dual-Gain Mode

Because of the non-overlapping sampling and conversion operations in the dual-gain mode, this coupling pathway does not cause crosstalk, thus allowing the usage of individual output lines without any multiplexing.

### 5.2. ADC Counter Distribution

The single-slope ADC counter is a custom-designed 2.8 GHz (1.4 GHz dual data rate), 12-bit gray counter placed inside reticle blocks A and G. Due to the large footprint of the die, we need repeaters periodically to distribute the ADC counter horizontally across the chip as well as vertically for each stacked analog SRAM. The analog SRAM cell is laid out at a pitch of 3.9 µm, allowing routing channels to be created (every 200 SRAM column) for vertically routing ADC counter buffers.

**Figure 9:**
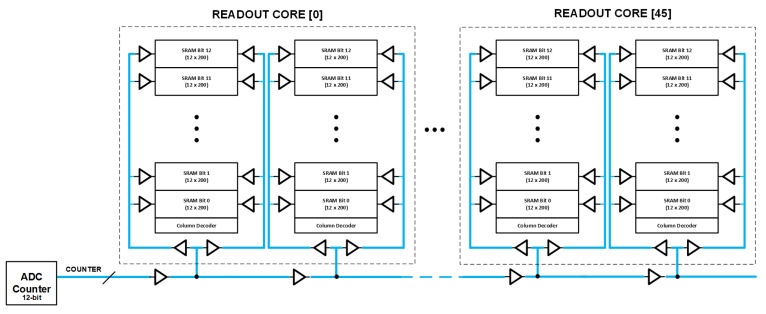
Conventional single-ended ADC counter distribution.

Figure 9 shows a conventional ADC counter distribution approach. One of the main issues in high-speed counter distribution over a long distance is the degradation of the duty cycle of counter bits quantified as differential non-linearity (DNL) of the ADC [13]. This DNL is primarily caused by a process mismatch between the NMOS and PMOS devices of the inverter buffers used in the ADC counter distribution causing duty cycle distortion. This issue can be more prominently seen in the slow–fast and fast–slow process corners of the technology node.

**Figure 10:**
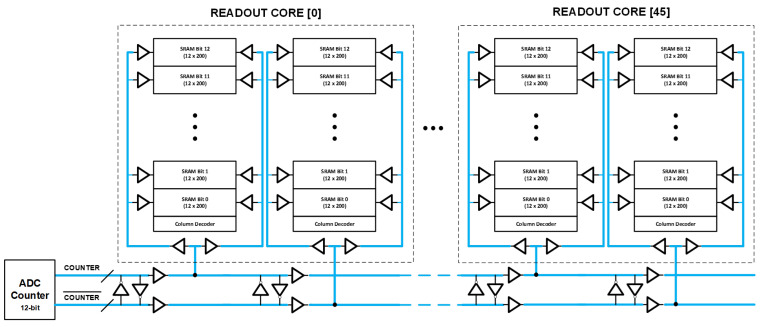
Proposed pseudo-differential ADC counter distribution.

To improve the accumulated DNL, we implemented a pseudo-differential ADC counter distribution (Figure 10). The main idea was to horizontally distribute the 12-bit gray counter along with its compliment (bit-wise NOT operation). Within the readout core, after every 200 SRAM columns, we flipped the connection of the vertical ADC counter distribution where it taps to the horizontal ADC counter distribution. Because of this periodic flip, any accumulation of duty cycle distortion (or DNL) is significantly reduced. The digital block in the readout core is pre-configured to compensate for this bit flip after every 200 SRAM columns.

Table 2 compares the simulated DNL of the single-ended ADC counter distribution vs. the proposed pseudo-differential ADC counter distribution at various spatial locations. Clearly, there is more than 5× improvement in worst-case DNL with our proposed implementation.

### 5.3. High-Speed Clock Generation and Distribution

The large amount of data generated by the sensor necessitates a high aggregate data rate—even with 92 output data ports, the required data rate is 5.6 Gbps (per port DDR), requiring a 2.8 GHz clock to be distributed to each of the data ports along the ≈8 cm horizontal chip edges. There are a total of 18 PLLs (one each in stitch blocks ‘A’, ‘C’, ‘G’, ‘I’, ‘B’, and ‘H’) generating an output clock at 2.8 GHz from a 50 MHz reference clock provided externally (in each of the stitch blocks).

The 2.8 GHz PLL output CMOS clock is converted into the CML domain (for common mode noise rejection) and distributed to all readout cores within the stitch block through a high speed CML clock distribution network. Two CML buffers in the clock distribution are separated by a readout core pitch of ≈1.72 mm, necessitating a very careful design and optimization of routing traces between the CML buffers.

As a starting point, we routed the CML traces in the top thick metal having the minimum resistance and least coupling capacitance to the substrate. The trace width and separation were carefully fine-tuned based on a post-layout simulation with an RLCK extracted model to minimize attenuation at the operating frequency. Increasing the number of CML buffers to reduce inter-buffer separation has a trade-off with power and device noise. We selected a T-shaped clock distribution and based on simulation results decided on 4 cascaded CML buffers in each of the left and right directions from the center (in stitch blocks ‘B’ and ‘H’).

The output of one CML buffer is AC-coupled into the input of the next stage buffer. This allows us to set a well-defined common mode voltage at every CML buffer input pair. It helps to increase robustness by suppressing any systematic or process mismatch causing any common mode imbalance, as well as any low frequency noise. One downside of this AC coupling approach is that there is an additional attenuation in the signal path due to the use of MOS capacitors for AC coupling. At the nominal operating frequency, the overall attenuation due to lossy trace and the AC coupling stage should be compensated for by the large signal gain of the CML buffer.

### 5.4. Horizontal Smearing

Horizontal smearing is one of the primary array crosstalk artifacts in any large CIS chip using column parallel readout structures [14,15,16]. This artifact occurs when the readout of one portion of the image has a global effect on components that are common to the entire readout. The classical manifestation of this is a very bright region in the image causing a disturbance extending horizontally from the bright region. Significant design effort went into minimizing the absolute value and curvature of this specific artifact.

The ramp generator output was buffered after every two readout units to reduce the ramp propagation delay and minimize the impact of any local ramp kickback causing smearing [17]. Additionally, all the ADC references were re-biased locally in every readout block to create a low-impedance net to the center of the array. This helped to locally restrict any reference disturbance caused by aggressor ADCs, which further helped to improve horizontal smearing performance.

**Figure 11:**
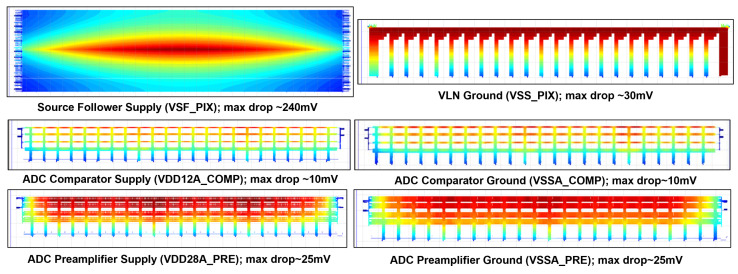
Generated thermal map from static IR drop simulation.

Another important ‘global’ factor causing smearing is the non-uniformity of pixel and various ADC supplies. Due to the sheer size of the sensor and the limited routing metal availability in this process, it is challenging to bring power to the pixel and various supply domains of the readout array. At the readout unit level, careful floor planning and layout optimization efforts went into minimizing the IR drop across the pixel array and readout blocks to improve supply uniformity. Among the various supply domains, a larger routing area (lower resistance to the pad) was allocated to supplies, causing more a significant impact on smearing performance based on simulation models. Finally, a comprehensive set of EMIR simulations were performed (Figure 11) for all the supply domains to ensure supply uniformity, thus minimizing the impact of smearing.

## 6. Results and Summary

**Figure 12:**
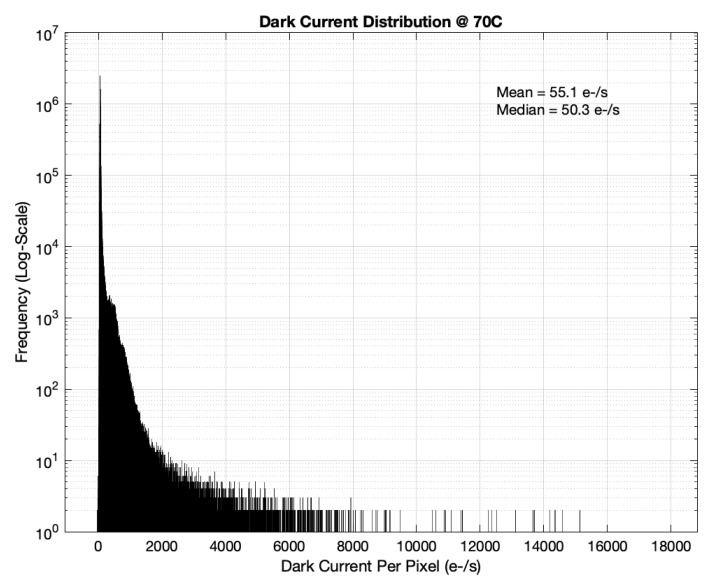
Measured dark current distribution.

**Figure 13:**
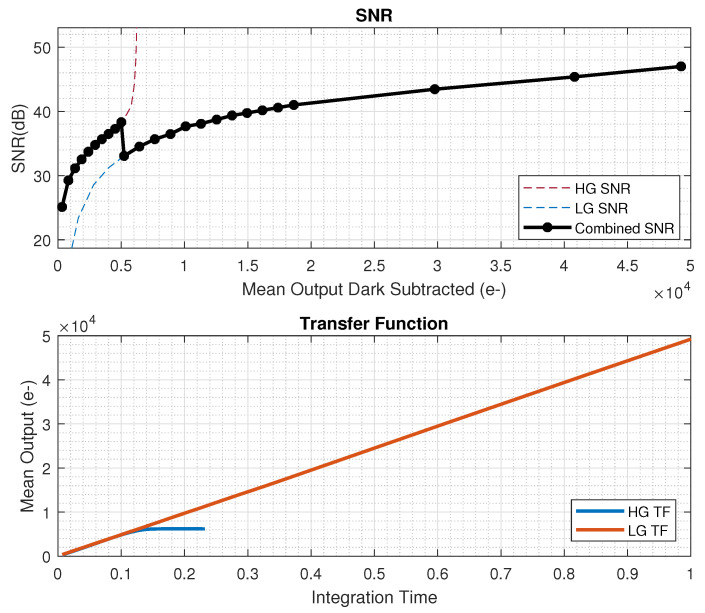
SNR and transfer function in HDR mode.

**Figure 14:**
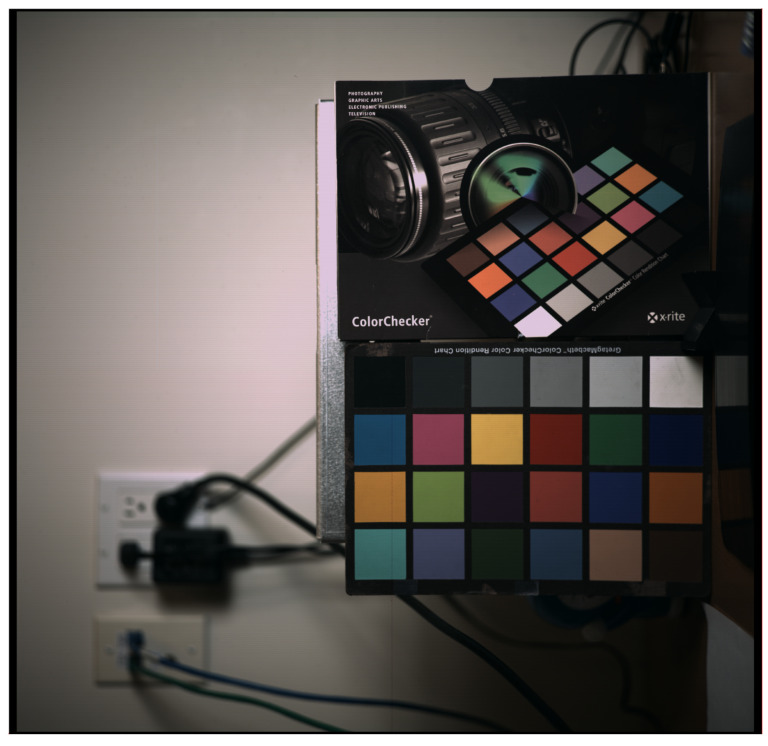
Full-resolution color image captured in single-gain mode at 120 FPS.

With the 4.3 µm dual-conversion-gain pixel, we measured a linear full well of 6600e- and 41,000e- in high- and low-conversion gain modes, respectively. The measured RMS temporal noise in the high-gain mode was 1.8e-, giving a composite dynamic range of 87 dB. The mean dark current of the sensor was 55e-/s as measured at a 70C junction temperature. A detailed dark current distribution is illustrated in Figure 12. The measured SNR plot for the HDR mode along with the high and low gain transfer functions are included in Figure 13. The overall power consumption of this CIS chip at 18 K resolution at 120FPS was 23 W. A comprehensive power breakdown is shown in Table 3, with the majority of power being burnt in ADCs, VLN, and high-speed blocks. Detailed specifications of the CIS sensor are summarized in Table 4, and a full resolution color image captured at 120FPS (single-gain mode) is shown in Figure 14.

Finally, a performance comparison of this large-format CIS chip with other publicly available state-of-the-art large-format image sensors is shown in Table 5. Our sensor has a 120× faster maximum frame rate compared to [18] in spite of reading a higher number of pixel rows with a larger pitch. This is achieved with a 2× better dark temporal noise, 9 dB better dynamic range, and more than 3× better dark current performance (assuming a dark current doubling factor of 7C). Compared to GMAX3005 [19], we have achieved a 12× faster maximum frame rate, >2× improvement in dark temporal noise, and 20 dB improvement in dynamic range with a >7× better dark current.

## Figures and Tables

**Table 1:** ADC crosstalk improvement (simulated): single-gain mode.

Aggressor–Victim (Victim Condition)	No Dynamic Shield	With Dynamic Shield
Ping0–Pong1 (Dark)	4.01 DN	0.25 DN
Ping1–Pong1 (Dark)	2.03 DN	0.18 DN
Ping0–Ping1 (Bright)	−16.5 DN	0.04 DN
Ping0–Pong1 (Bright)	−3.7 DN	0.3 DN
Ping1–Pong1 (Bright)	−1.7 DN	0.1 DN
Pong0–Ping1 (Dark)	0.76 DN	0.05 DN
Pong0–Ping1 (Bright)	−0.72 DN	0.01 DN

**Table 2:** ADC counter DNL improvement for single-ended vs. differential ADC counter distribution.

Spatial Location	Single-Ended (DNL)	Pseudo-Differential (DNL)
Readout Core[0] (first)	0.166 DN	0.111 DN
Readout Core[45] (last)	0.637 DN	0.112 DN

**Table 3:** Measured power consumption (blockwise distribution).

Block	Percentage Power Consumption
ADC Preamplifier and Ramp Distribution	37.41
VLN (Column Bias)	24.72
CML (Clock Distribution + Serializer + Output Driver)	16.03
Ramp Generator and Distributed Buffers	8.12
Analog SRAM	5.30
Digital Blocks	5.04
ADC Counter and Distribution Network	1.58
ADC Comparator	0.97
Row Logic	0.82
Voltage References	0.01

**Table 4:** Sensor specification table.

Paramater	Specification
Pixel Pitch	4.3 µm
Total Pixels	18,400(H) × 17,712(V)
Active Pixels	18,000(H) × 17,568(V)
Row Time	Single Gain: 5.5 µs, Dual Gain: 11 µs
Maximum Frame Rate	Single Gain: 120 FPS, Dual Gain: 60 FPS
ADC Resolution	12 bits (2.8 GHz count rate)
Linear QSat Full Well	High Gain: 6600e-, Low Gain: 41,000e-
Conversion Gain	High Gain: 150 µV/e-, Low Gain: 19.1 µV/e-
Total Temporal Noise	High Gain: 1.8e-, Low Gain: 13e-
Dynamic Range	87 dB
Image Lag	0.45e-
PRNU (ROI: 4000 × 3000)	0.8%
Dark Current	55e-/s (measured at 70C)
Total Sensor Power	23 W
Die Size	9.92 cm × 8.31 cm

**Table 5:** Performance comparison with state-of-the-art large-format CIS chips.

Parameter	This Work	Bogaerts et al. [18]	GMAX32152 [20]	GMAX3005 [19]
Technology	65 nm BSI	90 nm FE, 65 nm BE	-	-
Pixel Pitch	4.3 µm	3.9 µm	3.2 µm	5.5 µm
Pixel Architecture	5T non-shared dual gain	4T non-shared	-	4T with binning
Shutter Type	Rolling	Rolling	Global	Rolling
Total Number of Pixels	18,400(H) × 17,712(V)	26,456(H) × 15,072(V)	16,556(H) × 9200(V)	30,000(H) × 5000(V)
Active Area	77.4(H) mm × 75.54(V) mm	101.84(H) mm × 58.50(V) mm	53(H) mm × 29.4(V) mm	165(H) mm × 27.5(V) mm
Chip Size	83.1(H) mm × 99.2(V) mm	105.18(H) mm × 65.63(V) mm	59(H) mm × 35.2(V) mm	167.6(H) mm × 30.1(V) mm
Maximum Frame Rate	120 FPS (single gain), 60 FPS (HDR)	1 FPS	16 FPS	10 FPS
Effective Pixel Rate	39.11 GPix/s	0.4 GPix/s	2.44 GPix/s	1.5 GPix/s
Output Data	92 CML @ 5.6 Gbps (DDR)	24 LVDS @ 300 Mbps (DDR)	38 sub-LVDS @960 Mbps	120 LVDS @ 200 Mbps
ADC	12-bit SS ADC	14-bit SS ADC	12-bit SS ADC	16-bit SS ADC
ADC Counter Rate	2.8 GHz	150 MHz	-	-
Linear Full Well Capacity	41ke- (LG); 6.6ke- (HG)	31.5ke-	9.3ke- @ 1.4 × gain	23ke-
Conversion Gain	HG:150 µV/e-, LG:19.1 µV/e-	45 µV/e-	-	-
HDR Mode	Yes	No	No	Yes
Dark Temporal Noise	1.8e-	3.7e- at 1× gain	5e- @ 1.4× gain	3.94e-
Dynamic Range	87 dB (intrascenic)	>78 dB	>65.4 dB @ 1.4× gain	67 dB (intrascenic)
PRNU	0.8% (ROI: 4000 × 3000)	1%	-	-
Dark Current	55e-/s (70C)	95e-/s (60C)	1.4e-/s (30C)	<10e-/s (32C)
Power Consumption	23 W @120FPS full-resolution 12-bit	1.75 W @1FPS full-resolution 14-bit	<2.8W @16FPS full-resolution 12-bit	2.5 W @10FPS full-resolution 16-bit

## Data Availability

Not available.
